# Prize Essay on the Nature and Objects of Medical Science, and the Principles upon Which Its Study and Practice Ought to Be Founded

**Published:** 1844-07

**Authors:** 


Art. V.—*Prize Essay on the Nature and Objects of Medical Science, and the Principles upon which its Study and Practice ought to be founded.* By Philip Henry Williams, m.d.—*Edinburgh,* 1844. 8vo, pp. 52.

The contents of this essay do not, for the most part, require any special notice. Nearly one half of the essay is occupied with an hypothesis which is foreign to the subject announced in the title-page. It is however, ingenious, and if expatiated upon, might possibly have made a better thesis than that of which it now forms a somewhat incongruous portion. Such hypotheses, though inadmissible in practical writings, are not to be objected to in an academical thesis, which affords fair ground for practical disquisition, for the exercise of ingenuity, or for the display of learning according to the inclination of the writer.

Dr. Williams is of opinion that the periodicity of intermittent fever may be accounted for on the supposition of the gradual decomposition of the morbific poison, and that febrifuge remedies cure the disease by acting as antiseptics on the poison. We extract the following curious passage :

"The hypothesis, however, that appears to the writer most satisfactory to account for the intermission is this, viz. that the malarious matter *when inhaled,* is in a *graduated* state of decomposition, and that, under the circumstances supposed, it will operate at distinctly graduated intervals. . . . . . . . . The *fact* of regular intervals occurring is one decided argument in favour of some graduation in the operation of their cause, vegetable matter. We know that decomposition is, beyond all question, a gradual process, and it is probable that a quantity of marshy air would contain matter in different degrees of decomposition. If this be granted, no argument is required to prove that, on the supposition of the most decomposed matter being the most poisonous, the operation of such a graduated substance would occur at pretty regular intervals, precisely the same *relative* decomposition being maintained between the different parts, throughout the whole operation. Thus if the dots . . . . . . are supposed to represent the matter in its most poisonous state, and if $\begin{matrix}A & B & C & D & E\\ O & O & o & o & o\end{matrix}$ represent the remainder it is clear that, when E had assumed the dotted or poisonous forms, A B C D would be in the precise state in which B C D E were previously. It may be replied, 'then of course the operation would be *continuous.*' But it has been stated that the reaction following the operation of the first poisonous attack might fairly be supposed to arrest the whole process, and therefore E would remain in the state of E so long as the reaction continued; and, as soon as it ceased, E would proceed into the state of the dots indicating the poison, and D during that change, would assume the state of E, and so again be ready to pass into the dotted condition, when the next reaction ceased. And this leads to the argument in favour of the reaction causing an arrest of the poisonous development. It is this: if no reaction occurs, a *continuous* operation is the consequence; the malarious matter has full play upon the system, and comparatively little good can be done. We have in fact a case of continued or typhoid fever; the most decomposed matter is not eliminated, and a successive action is kept up on the nervous system, which effectually maintains its depression, and the depression of the circulation." (pp. 35-6.)

There is, to our mind, something rather Shandean and amusing in the mode of illustration here adopted by the author; but we much fear that some thick-headed people will not understand his meaning. Dr. Williams is inclined to similar views respecting the operation of animal poisons, in accounting for which, he advocates the cause of gradual decomposition of animal matter against that of the gradual development of ova. But we cannot further extend our notice of Dr. Williams's thesis. It is clever, and indicates some originality of mind. As it gained a gold medal from the University of Edinburgh, the author had no option but to print it in conformity with the rules of that body, otherwise its contents might perhaps scarcely be deemed of sufficient importance to render their publication desirable.

